# Ebola virus disease surveillance in the absence of a confirmed case; the case of the Rwenzori region of Uganda

**DOI:** 10.11604/pamj.2020.37.137.22957

**Published:** 2020-10-08

**Authors:** Emmanuel Angmorteh Mensah, Annet Kisakye, Samuel Ofori Gyasi

**Affiliations:** 1 Disease Control Unit, Kwatire Polyclinic, Sunyani West Municipal, Bono Region, Ghana,; 2 Department of Immunization, Vaccines and Biologicals, World Health Organization Country Office, Kampala, Uganda,; 3 Health Information Department, Western Regional Health Directorate, Takoradi, Ghana

**Keywords:** Ebola virus disease, Kasese district, surveillance activities, preparedness, Rwenzori region, points of entry

## Abstract

**Introduction:**

the August 2018 ebola outbreak in the Democratic Republic of Congo turns out to be second largest outbreak of ebola in public health history. The response to the outbreak which would have halted wider spread to neighboring countries failed. Hence, high risk districts in Uganda initiated preparedness activities in the wake of a possible inflow of cases. This study was therefore designed to identify, describe and assess surveillance activities and preparedness in the Kasese, Ntoroko and Bundibugyo districts of Uganda.

**Methods:**

the study employed the mixed method approach. The qualitative arm involved the use of participant observation to describe surveillance activities that were carried out as part of the ebola preparedness surveillance in the high-risk districts. The quantitative arm included assessment of 102 health facilities on ebola virus disease preparedness with a WHO standard checklist hosted on the Open Data Kit software. Descriptive statistics were performed using STATA (version 14).

**Results:**

the study showed that high risk districts employed numerous interlocking public health emergency activities which included readiness assessment, risk mapping and temperature-based screening for ebola at points of entry. Most health workers (91.18%) could correctly state the case definition of ebola although only 56.86% of them were trained on ebola surveillance.

**Conclusion:**

health worker knowledge on ebola virus disease case definition was high but training and logistics were inadequate. Continuous efforts are required to sustain health workers knowledge on ebola surveillance through trainings and supportive supervision whiles addressing gaps in the operation of ebola screening posts.

## Introduction

Ebola virus disease perhaps is one of the greatest public health threat in recent times having caused over fifteen outbreaks in eleven countries since 2011 with the deadliest of which occurred in some countries across West Africa [1]. In ebola West Africa, a total of 20,035 confirmed and probable cases of ebola virus disease (EVD) were reported in Sierra Leone, Liberia, and Guinea from December 2013 to August 2015 [2]. The current outbreak (August, 2018) in the Democratic Republic of Congo (DRC) is characterized with population movement in highly densely populated areas; weak infection and prevention control practices in many health facilities; complex political environment; and the ongoing unstable security situation, which led to the murders of both international and local health workers [3]. As at mid-January 2020, at total of 3395 cases were detected from Ituri, North and South Kivu provinces. The outbreak has so far recorded a case fatality rate of 65.83% [4]. With the recent less difficulty in travelling and trade, geographical distance is no longer a major hindrance in transmission of infectious diseases [5]. The outbreak has therefore posed a huge challenge to the health security of neighboring countries including Uganda considering the possibility of geographical spread [6]. Despite the glaring threat of the outbreak that was only 100 metres away from the Rwenzori region of Uganda, usual cross border activities including trade and social activities continued normally with no interruption by the principles of the International Health Regulation [7]. High risk districts in the Rwenzori region and Uganda as a whole therefore initiated preparedness activities in the wake of a possible inflow of cases. The region has a setting in which infectious diseases can be easily spread and transmission sustained. Therefore, preparedness using available public health resources was the highest priority [8]. Adequate preparations are required to control communicable diseases such as ebola that does not only affect individuals but also spread rapidly through communities. Prompt identification and response efforts are possible when there is effective link among communities, public health facilities and laboratories [9]. Lack of preparation leads to untimely identification of cases thereby hindering rapid and effective implementation of control efforts required in hampering the establishment of an outbreak [10].

Preparedness in infectious disease control depends on many factors such as robust disease surveillance systems, reliable health information, prevention, diagnostic, and treatment services, financing, and strong political commitment and skilled health professionals, who should be valued and protected everywhere, to act as the first line of defense of individual health security [7]. The World Health Organization (WHO) Uganda country office therefore led [11] other technical agencies such as the Infectious Disease Institute (IDI), Uganda Red Cross and UNICEF to support Ministry of Health (MOH) staffs at the district and health facilities in order to implement activities aiming at preventing, detecting, and responding to any imported or indigenous case since border controls cannot stop the international spread of disease [12]. Nonetheless, it not known how the activities by the MOH and its partners adequately prepared health workers, community members and health facilities towards detection and response to outbreak. Additionally, Kagando Hospital in the Kasese district in June 2019 suspected a patient to be having ebola disease. Laboratory finding prove positive for ebola virus. Two additional family members who were in contact with the initial case later developed the disease. Were it not for the early identification, the disease would have established leading to major outbreak. It is stipulated that the prompt identification of the ebola cases was due to rigorous surveillance activities and preparedness of health facilities in the Rwenzori region. This study was therefore designed to identify and describe the surveillance activities that were in place in the Rwenzori region prior to the case detection and also assessed the level of preparedness of health facilities in the high-risk districts. The study therefore fills the literature gap as far as EVD preparedness surveillance is concerned by sharing observations and provide data pertaining magnitude of preparedness of health facilities.

## Methods

**Study design:** observational and descriptive studies on systems put in place to ensure adequate preparedness for ebola virus diseases in the wake of possible spillover from the Democratic Republic of Congo to Rwenzori region of Uganda. The study used both qualitative and quantitative approach to describe surveillance activities/procedure that were carried as part of the preparedness and also assessed health facilities with respect to knowledge of staff on EVD, logistics and Standard Operating Procedures (SOPs) availability and also Alert reporting.

**Study site description:** the Rwenzori region of Uganda is located within the western part of the country. It is made up by districts around the Mountain Rwenzori which stretches through the region into the Democratic Republic of Congo (DRC). Five districts in the region were classified as high-risk district for ebola due to their proximity form the ongoing outbreak. These districts are Kaborole, Bundidugyo, Ntoroko, Kasese and Buyangabu.

**Study population:** the qualitive aspect of the study observed activities and individuals within selected districts for EVD preparedness activities including individuals who cross into those districts from DRC for the purpose of healthcare, trade and other social and economic activity with emphasis on health care professional with various agencies engaged in surveillance activities during preparedness phase for ebola. The quantitative aspect assessed health facilities preparedness within the study area.

**Exclusion and inclusion criteria:** although five high-risk districts form part of the Rwenzori region, districts without Points of Entry (PoE) or borders directly with DRC were excluded from the study. The study therefore focused on Kasese, Ntoroko and Bundibugyo only.

**Sampling and sample size:** all health facilities within the districts were assessed. Data drawn from the ODK server for the period of August 2018 to May 2019 showed that, 93 health facility based EVD assessments were performed in the Kasese district, 32 in Bundibugyo and 29 in Ntoroko district. It was observed that some health facilities were assessed repeatedly at difference times within the period of study. The study ignored earlier assessments that were done in case facility had more than one entry. The most recent assessment for a health facility was used for the analysis since it is the true state of the facility prior to exiting of preparedness to response phase. The most recent assessment in each health facility in the districts were 77 for Kasese, 15 for Bundibugyo and 10 for Ntoroko district, making a total of 102 for the study.

**Data collection procedure:** mixed method approach was used to collect data. The qualitative arm involved participant observation (observations made by researchers on the field during the period of preparedness documented by way of reports and field notes). The observations with regard to surveillance activities were scripted with reference to reports and personal notes on an observational checklist. The individual scripts of the researchers on surveillance were then merged. The quantitative arm includes the use of available data captured with the Open Data Kit (ODK) on alerts, health facilities preparedness and Points of Entry´s (PoEs) screening data. The health facility assessment was done with health facility EVD preparedness assessment checklist. A standard tool by the World Health Organization hosted on Open Data Kit (ODK).

**Data analysis:** data saved on the ODK server was extracted and exported in excel format. The data was cleaned and checked for accuracy and completeness and then imported to STATA (STATA 14) for analysis and presentation in charts and graphs. Descriptive statistics were performed with STATA (STATA 14) which involved cross-tabulations to show proportions of indicators/variables. Key indicators of EVD preparedness assessed were availability of EVD SOPs, training of health facility Surveillance Focal Persons on EVD surveillance and knowledge on EVD case definition, evidence of active search for EVD. The qualitative analysis was achieved by the principal investigator reading the narrative on the observational checklist scripted by the individual researchers. Thematic concepts in ebola surveillance as observed by researchers were coded and aggregated in categories by combining like information. The combined findings on the surveillance procedure in high-risk districts were agreed upon by all the researchers.

**Ethical considerations:** permission was obtained from the appropriate authorities including the World Health Organization Country Office and the Ministry of Health in order to access and use available data/records. The activities described and data analyzed were part of the preparedness interventions of the Ministry of Health of Uganda/World Health Organization Country Office respond to possible influx of ebola from the Democratic Republic of Congo. We did not use any confidential data and did not disclose any unauthorized names in our report.

## Results

**Ebola preparedness surveillance activities in the Rwenzori region of Uganda:** the EVD preparedness in the Rwenzori region combined various public health emergency strategies and activities under the coordination of District Ebola Task Force (DETF). Two subgroups within the DETF spearheaded surveillance activities in the districts. The teams were the surveillance subcommittee and the Rapid Respond Team (RRT). Below were the findings with regard to surveillance procedure or activities that were undertaken in the absence of a confirmed case.

**Readiness assessment:** the initial activity was a readiness assessment by a national team deployed by the Ministry of Health. The team from the national level used the World Health Organization´s EVD readiness assessment tool to examine the level of readiness of the high-risk districts to detect and manage ebola cases in the event of any inflow. The team also supported the districts to identify actions to be performed to ensure operational readiness including the establishment of the district ebola task force. The readiness assessment found the following surveillance gaps in the districts. Lack hotline in place for reporting of cases, inadequate case investigation forms and standard case definitions and inadequate training in EVD surveillance.

**Risk mapping:** the risk mapping majorly focused on Uganda - Democratic Republic of Congo border population movements, reasons for their movements, border crossing points and major destinations in either countries. The risk mapping identified points of entry. The points of entry were further classified based on the average number of individuals who crosses on daily basis and nearness to a corresponding community in the Democratic Republic of Congo in which cases are occurring. The outcome of the risk mapping aided prioritization of activities.

**Screening at points of entry (PoEs):** screening facilities were set up at points of entry. Trained volunteers mount these facilities and ensures individuals entering Uganda washes their hands with 0.05% chlorinated water, dip their foots in 0.5% chlorinated water and temperature checked with infra-red thermometers within reasonable distance. Individuals with temperatures of 38°C and above were detained for about thirty minutes for verification of the high temperature. Detained individuals with high temperatures were considered as alerts case. The volunteers at the screening facilities then informs the rapid response team for assistant. The team upon reaching the site conducts further enquiries and transfer the patient to the Ebola Treatment Unit (ETU) for investigations and management. The screening facilities were organized in four units, the hand washing, temperature checking, data recording and case holding zones. Not all identified crossing points were conducting screening due to inadequate logistics and manpower. Some crossing points were seen to pose less threat according to the risk mapping. Table 1 shows the proportion of PoEs that were conducting screening activities during the preparedness phase per district.

**Table 1:** proportion of points of entry conducting temperature-based screening for ebola during preparedness phase

District	PoEs Identified	PoEs Screening for Ebola	Percentage (%)
Kasese	31	5	16.13
Bundibugyo	16	5	31.25
Ntoroko	27	18	66.67
**Total**	**74**	**28**	**37.84**

**Capacity building for ebola detection and active search:** formal training and Health facility-based capacity building in ebola detection were used. Training on ebola surveillance and contact tracing for health facility heads and surveillance focal persons. The training equipped participants on case definition of ebola and order of reporting identified cases. The district surveillance teams conducted health facility-based capacity building of health workers on identification of ebola and the appropriate measures to implement when a case is detected.

**Community based ebola surveillance and sensitization:** another surveillance activity identified was active search for ebola cases in communities. The focus of the community visit was the Village Health Teams (VHTs), Community Development Officers (CDOs), Political and Opinion leaders. They were sensitized on the disease and asked for the presence of any case in the community. Follow ups were conducted whenever a case was reported. Organize groups, markets and schools were also sensitized and EVD posters with hotlines for reporting suspected cases were also distributed. The district surveillance teams exchange contacts with VHTs and community leaders for easy information sharing and response to alerts.

**Alert response and case investigation:** there were four (4) main sources of alerts. Alerts which came from the points of entry screening, health facilities, rumours (unofficial/indirect information) and VHTs and community leaders. The Rapid Response Team (RRT) which is composed of surveillance and case management personnel were deployed to the locations when an alert was given. The RRT assess the alert case including travel and exposure history from the health facility, screening point or community. Cases that met the case definitions were transported to the Ebola Treatment Units (ETU) for case-based investigation. During the preparedness phase, a total of 77 alerts were investigated and geocoded using ODK. Table 2 shows the demographic characteristics of the alerts. Bundibugyo district recorded the highest number of alerts.

**Table 2:** socio-demographic characteristics and outcome of alerts recorded during ebola preparedness in the Rwenzori region

Variable	Category	Frequency (N=77)	Percentage (%)
**Alerts Reported by Districts**	Bundibugyo	42	54.55
	Kasese	18	23.38
	Ntoroko	17	22.08
**Sources of Alerts**	Community	22	28.57
	Health Facility	34	44.16
	Screening Points (PoEs)	21	27.27
**Age of Alert cases**	≤14 years	20	25.97
	15 - 64	54	70.13
	65+	3	3.90
**Occupation**	Trader	5	6.49
	Child/Pupil	25	32.47
	Farmer/Hunter	19	24.68
	Others	26	33.77
	Unemployed	2	2.60
**Travel History to DRC**	Yes	26	33.75
	No	43	55.84
	Travel history not defined	8	10.39
**Alerts with Specimen collected**	Specimen collected	68	88.31
	Specimen not taken	9	11.69
**Outcome of case**	Survived	72	93.51
	Died	5	6.49

**Preparedness of health facilities prior to confirming of ebola in Uganda:** from Table 3, the study showed only 30.39% of health facilities were having ebola virus disease Standard Operating Procedure guideline. Majority 56.86% [58/102] of respondent were trained on ebola surveillance. Most health workers could correctly state the case definition of ebola. Close to 50% of the health facilities had no evidence of active search for EVD in outpatient department registers. Eighty eight percent (88%) of health facilities had poster or information, education and communication materials on ebola surveillance at vantage points to educate health facility users.

**Table 3:** preparedness level of health facilities prior to detection of ebola in Uganda in June 2019

Variable	Category	Frequency (N = 102)	Percentage (%)
**Level of Health Facility Visited**	District Hospital	1	0.98
	General Hospital	4	3.92
	Health Centre IV	10	9.80
	Health Centre III	56	54.90
	Health Centre II	30	29.41
	Clinic	1	0.98
**EVD SOPs**	Available	31	30.39
	Unavailable	71	69.61
**HF Surveillance Focal Persons Trained on EVD**	Trained	58	56.86
	Not Trained	44	43.14
**Knowledge on EVD case Definition**	Knowledgeable	93	91.18
	Not Knowledgeable	9	8.82
**Evidence of Active Search for EVD**	Yes	53	51.96
	No	49	48.04
**Display of EVD Poster**	Displayed	88	86.27
	Not Displayed	14	13.73
**Alert Reporting**	No Unreported Alert	100	98.04
	Unreported Alert	2	1.96
**Case Investigation Form**	Available	7	6.85
	Unavailable	95	93.14
**List of Community Informants**	Available	81	79.41
	Unavailable	21	20.59

## Discussion

The August 2018 ebola outbreak, the tenth to hit Democratic Republic of Congo (DRC) turns out to be second largest outbreak of ebola in public health history. The response to the outbreak which would have halted wider spread to neighboring countries failed [13] but Uganda has been commended for the prompt identification and swift response to the ebola outbreak in June 2019 [14] in the Kasese district. Notable among the individuals who were pleased was the US secretary for Health [15]. The findings of the study avails significant information on ebola preparedness in high risk countries with no cases along counties with active outbreak.

**Ebola preparedness surveillance activities in the Rwenzori region of Uganda:** readiness assessment being the foremost activity was needful since it helped to identify gaps in the high-risk districts. The World Health Organization (WHO) standard tool; ebola virus disease consolidated preparedness checklist [16] gave a sense of direction in the initiation of preparedness activities. The tool however was not designed to provide aggregate score for the districts assessed. Ebola is a disease with onset of high fever and spread through contact [17]. With modern technologies and advancements in health care, temperatures can be measured without any physical contact. In that principle, screening post were established at selected points of entry. In coming individuals were checked for high temperatures ranging from 38°C. But could high temperature be solely used as yardstick for ebola investigations. The basic symptom in most diseases is high temperature. In that regard, there should be more standard questionnaire for further assessment for ebola in individuals with high fever detected at ebola screening points. Key indicators may include contact history, duration of fever and previous or current sickness. The weakness of this method of ebola screening and identification is the fact that infected individuals do not present with fever during incubation period. Communities may still record cases from individuals who were actually screened prior to entry but were incubating the disease. Scientific predictions have it that the efficiency of entry screening to detect a single case range from 0.004% to 0.009% even in advance countries [18]. Both sensitivity and specificity of temperature-based screening for ebola at points of entry remains questionable. Also, both nationals of Uganda and foreigners who were identified as alerts or suspect were transported to designated Ebola Treatment Units (ETUs). Considering it that foreigners with usual place of residence in DRC have a higher risk of ebola than Uganda nationals who are returning after a short period in DRC, some schools of thoughts have it that transporting individuals from DRC with fever to ETUs in Uganda is tantamount to deliberate case importation. In their view, it is better to return individuals with fever to their hometowns. This will prevent cases from entering the country. The option to return visitors with fever back rather than transporting them to ETUs in Uganda is justifiable in a way. Ebola being a highly infectious disease could establish in new places with the least mishandling. So, the safest option would have been returning visitors to settings where there are already cases. Also, in cases where minors were considered as alerts, the challenge remains lack of caregivers to consent. Will it therefore be appropriate to return such a minor? Noncompliance was also an issue at the crossing points. In coming individuals were not ready to avail themselves for the screening. Screening points such as Mpondwe in Kasese district and Busunga in Bundibugyo district had a military presence to ensure complaints. Ebola surveillance points of entry therefore goes beyond health sector alone but a collaborative effort by health, police, immigration and military. Many gaps exist in the operations of temperature-based screening points for ebola which must addressed.

Training is a way of equipping professionals with needed skills to handle a situation [19]. Selected health workers were trained on ebola surveillance and contact tracing. These has been a traditional approach in public health emergencies to address knowledge gaps as it was done in Sierra Leone ebola outbreak [20]. In Liberia, health facility-based approach was used to successfully train health workers on infection prevention and control in even hard to reach areas [21]. Formal training could have delayed activities and reduce the number of staff who would have been trained (selected few). Facility based approached made it possible to reach wide range of health workers. Most public health intervention in recent times are community based. Surprisingly, only 33.75% of alerts recorded had a travel history to DRC. Investigation of alerts without travel history shows that the surveillance system was not skewed to incoming cases only but equally focused on detection of cases from within. It is not out of order since Uganda has previously recorded indigenous case in Gulu [22] and Bundibugyo [23].

**Preparedness of health facilities prior to Kasese outbreak in Uganda:** from Table 2, health facilities were the highest sources of alerts with 44.16% of all alerts. This possibly justifies the effectiveness and importance of the health facility-based capacity building on ebola detection. However, the assessment of health facilities determined the availability of Standard Operating Procedures (SOPs) and surveillance job aids; guidelines on handling ebola suspects and alerts in the high-risk districts. Only 31/102 (30.39%) were having SOPs on ebola virus disease as shown in Table 3. The availability of SOPs can be said to be suboptimal. All health facilities should have the SOPs and surveillance job aids that can be referred to in case of any alert. The strategic response plan for the ebola virus disease outbreak in the provinces of North Kivu and Ituri of the Democratic Republic of Congo recommends the provision of surveillance guidelines to all facilities in risk zones [24]. The data also shows that significant proportion (43.14%) of health facility surveillance focal persons were not formally trained on ebola surveillance. Bemah *et al*. in their study on strengthening healthcare workforce capacity during and post ebola outbreaks in Liberia showed that training improves the knowledge and confidence of health staff. Improve knowledge and confidence significantly leads to reduction in health care workers infection and reduction response time to subsequent public health events [25]. Despite the lack of formal training on ebola for majority of health facility surveillance focal persons, 91.8% of them were knowledgeable on ebola case definition and detection. They could tell the standard case definition and basic clinical presentations of the disease. The knowledge level on ebola among health workers is significantly high as compared to community members. The study of Kankya *et al*. in the Bundibugyo district showed that slightly over 51% community had knowledge about EVD [26]. About 80% of health facilities had updated list of community informants sensitized on ebola detection. The list is very helpful in information sharing and follow up. Similarly, preparedness activities to enhance ebola virus disease surveillance and prevention in counties without confirmed cases in rural Liberia did not leave communities out [27]. Okware *et al*. in their study “Managing ebola from rural to urban slum settings: experiences from Uganda” reveal that community leadership and mobilization was very important in early case detection and isolations well as contact tracing and public education [22].

## Conclusion

Whereas there were no confirmed cases of ebola in the Rwenzori region of Uganda, high risk districts implemented varied public health surveillance interventions similar to surveillance activities in outbreak settings. The prompt identification and swift response to the ebola outbreak in June 2019 in the Kasese district could be associated with high health workers knowledge on case definition and detection of ebola. That notwithstanding, several gaps existed in the operations of temperature-based screening points for ebola which must addressed. Health facilities played a major role in identification of alerts amidst inadequate training and resources such EVD surveillance guideline. Continuous efforts are recommended to sustain health workers knowledge on EVD surveillance through trainings and supportive supervision whiles addressing gaps in the operation of ebola screening posts.

### What is known about this topic

- With the recent less difficulty in travelling and trade, geographical distance is no longer a major hindrance in transmission of infectious diseases such ebola;
- The outbreak in DRC posed a huge challenge to the health security of neighboring countries including Uganda considering the possibility of geographical spread;
- Ebola preparedness surveillance involves non-contact temperate based screening at points of entry (PoEs).

### What this study adds

- Health worker knowledge on EVD case definition was high in the high-risk districts which was key in the prompt identification of the Kasese outbreak;
- Thirty point thirty-nine percent (30.39%) of health facilities were having ebola virus disease standard operating procedure guideline;
- Eighty eight percent (88%) of health facilities had poster or information, education and communication materials on ebola surveillance at vantage points to educate health facility users.
